# Dual polymer networks: a new strategy in expanding the repertoire of hydrogels for biomedical applications

**DOI:** 10.1007/s10856-019-6316-9

**Published:** 2019-10-09

**Authors:** Shathani Nkhwa, Evren Kemal, Neelam Gurav, Sanjukta Deb

**Affiliations:** Centre for Oral Clinical and Translational Sciences, Faculty of Dentistry, Oral & Craniofacial Sciences, Guy’s Hospital, Floor 17, Tower Wing, London Bridge, London, SE1 9RT UK

## Abstract

Inspired by the double network hydrogel systems we report the formulation of dual networks, which expands the repertoire of this class of materials for potential biomedical applications. The tough dual network hydrogels were designed through sequential interpenetrating polymer formation, applying green chemistry and low-cost methods, devoid of any initiator-activator complexes that may pose risks in biomedical applications. The dual networks were synthesized in two steps, firstly the water soluble poly(vinyl alcohol) was subjected to cryogelation that formed the first network, which was then expanded by intrusion of a dilute solution of sodium alginate and complexed with a solution of calcium chloride under ambient conditions and further freeze-thawed. These hydrogels are flexible, ductile and porous with the ability to absorb and retain fluids as well as possess the versatility to easily incorporate biological molecules/drugs/antibiotics to be applied in tissue matrices or drug delivery systems. The dual network hydrogels can be tailored to have varying mechanical properties, shapes, size, thickness and particularly can be made physically porous if required, to suit the users intended application.

## Introduction

The ability of hydrogels to absorb water and biological fluids confers similarity with natural tissues that clearly make them an important class of materials for biomedical applications [1–3]. Hydrogels have thus received widespread interest in drug delivery, wound healing and tissue engineering applications, owing to their structural similarity to the extracellular matrix (ECM) and porous framework. Although they are used as drug delivery carriers, scaffolds for tissue engineering, absorbents and wound dressings, their weak mechanical properties, friability and low cell adherence limit their applications. For instance, hydrogels are suited for wound dressings because they provide a moist cool environment allowing exchange of nutrients [4] that can lead to a marked reduction in pain, ease and accelerate process of healing, however poor mechanical integrity leads to adverse patient compliance [5]. Examples also include the limited use in cartilage tissue engineering and gastric drug delivery due to the lack of survival and low toughness of the networks. Thus, engineered constructs should exhibit stress–strain responses comparable to the tissues they are intended to replace or provide structural support as the tissue regenerates whilst fulfilling the volume maintenance function [6, 7]. Suitable fluid diffusion and degradation properties are also required, which directly translate to mass transport properties of the hydrogel.

The current trend of creating tough hydrogels includes double network (DbN) gel formation which, essentially consist of two interpenetrating networks (IPN) with contrasting structures, one a densely cross-linked, brittle network of low concentration and the other a lightly cross-linked ductile network of high concentration [8, 9]. However, one of the requirements to form such networks is that the first network needs be a strong polyelectrolyte, which limits the different polymers that can be used. Secondly, the second monomer is polymerised in the first network thereby the presence of initiators-activators are inevitable in these networks that adversely affect performance in a biological environment. More recently, a molecular stent method has also been reported by Gong et al [10], proposing a more general method of toughening hydrogels. Unlike DbN gels, the method involves using a neutral hydrogel to create the first network and then by swelling it in a linear polyelectrolyte the first network is expanded akin to a stent [11]. This occurs as the polyelectrolyte functions as a dangling chain that produces a high osmotic pressure, which leads to high swelling and rigidity of the neutral gel.

Aiming to overcome some of these limitations, we employed the interpenetrating hydrogel design rationale to expand the repertoire of hydrogels for potential biomedical applications. In this paper we describe the formation of dual networks (DN) to create tough interpenetrating hydrogels. The formation of these networks is carried out sequentially, exclusively using green chemistry by employing physical crosslinking methods avoiding any toxic chemicals that make them highly desirable for medical use. These networks are analogous to double networks, where the first network is formed using a neutral, water soluble polymer, then crosslinked, followed by swelling in the second water soluble polyelectrolyte polymer, which can be chelated and then subjected to crosslinking. Hence, only polymers are utilized and a new polymer is not synthesized by entrapping the monomers in the first network unlike the double (DbN) or molecular stent networks. This obviates the use of any initiators, activators or crosslinking agents that may adversely affect the safety in use as biomaterials. This method also enables to overcome the usual lack of strength, toughness and tendency to rupture that is commonly associated with hydrogels.

## Experimental section

### Materials

Poly (vinyl alcohol) (PVA) (Merck, UK); [145,000 > kDa, 98%hydrolysis], Sodium alginate (Fisher Scientific) and Calcium chloride (Merck UK). Distilled water was used as the solvent. All materials were used with no further purification. All concentrations were calculated by weight to volume (w/v) in distilled water unless stated otherwise.

### Methods

#### Preparation of PVA films

Aqueous solutions of PVA were prepared at concentrations of 10% (PVA10) and 20% (PVA20) by heating the granules at 121 °C until fully dissolved. Solutions were allowed to cool down and poured into moulds before subjecting to various cycles of freeze drying (1, 2 and 3) for 24 h and thawing, to obtain PVA10-1FT, PVA10-2FT and PVA10-3FT and PVA20-2FT [12].

#### Preparation of the dual network of PVA/alginate films (DN)

The freeze thawed PVA10 hydrogel films were then allowed to swell to equilibrium in a solution of 2% sodium alginate (SA). The SA soaked PVA hydrogel films were then placed in 10% CaCl_2_ to allow chelation. The hydrogels were then separated into two groups for further physical crosslinking by (a) 1 freeze-thaw cycle (1FT) and (b) air-drying (AD) + freeze thawing (1FT) (AD+1FT).

#### Differential scanning calorimetry (DSC)

A Perkin Elmer Jade series differential scanning calorimeter was used to determine the thermal properties and Perkin Elmer Jade series software to process raw data. 10–20 mg samples were carefully placed and sealed in aluminium pans (Perkin Elmer). The scans were carried out with reference pan calibrated using Indium under a Nitrogen atmosphere. Two cycles of heating and cooling were carried out, starting from 0 to 250 °C followed by a cooling cycle to 10 °C at a rate of 10 °C per minute. The glass transition temperature (T_**g**_ °C) and melting temperature (T_**m**_ °C) were calculated using the Pyris Jade DSC (Perkin Elmer) software.

#### Fourier transform infrared (FTIR) spectra

FTIR was carried out using a Perkin Elmer spectrum FTIR spectrometer. ATR-FTIR spectra were obtained in the wavenumber range of 650–3500 cm^–1^ with 4 cm^−1^ resolution.

#### Equilibrium water content (EWC) and degree of swelling

The EWC and degree of swelling of the xerogels were determined using the following equations:

$${\mathrm{EWC}} = \frac{{{\mathrm{Ws}}-{\mathrm{Wd}}}}{{{\mathrm{Ws}}}} \times 100$$

$${\mathrm{Degree}}\,{\mathrm{of}}\,{\mathrm{Swelling}} = \frac{{{\mathrm{Ws}} - {\mathrm{Wd}}}}{{{\mathrm{Wd}}}}$$

Where Ws is hydrated weight when swollen, and Wd the dry weight of xerogel prior to swelling.

$$Weight\,swelling\,ratio\,\left( {SR} \right) = \frac{{{\mathrm{weight}}\,{\mathrm{of}}\,{\mathrm{the}}\,{\mathrm{swollen}}\,{\mathrm{hydrogel}}}}{{{\mathrm{weight}}\,{\mathrm{of}}\,{\mathrm{the}}\,{\mathrm{dry}}\,{\mathrm{hydrogel}}}}$$

The gel fraction was defined as the ratio of the dried gel mass weight to the initial mass weight of the polymer.

$${\mathrm{Gel}}\,\% = \frac{{{\mathrm{final}}\,{\mathrm{weight}}\,{\mathrm{of}}\,{\mathrm{dry}}\,{\mathrm{gel}}}}{{{\mathrm{initial}}\,{\mathrm{weight}}\,{\mathrm{of}}\,{\mathrm{dry}}\,{\mathrm{gel}}}} \ast 100$$

The following equations were used to determine the nature of diffusion of water into the hydrogels.

$$\frac{{{\mathrm{Mt}}}}{{{\mathrm{M}}_\infty }} = 2\left( {\frac{{Dt}}{{\pi l2}}} \right)^{1/2}$$

Where Mt and M∞ denote the amount of solvent diffused into the gel at time t and an infinite time (equilibrium) respectively. The slope s is obtained from the slope of a straight line of the plot M_t_/M_∞_ against t^1/2^ where *l* is the thickness and D is the diffusion coefficient.

$${\mathrm{s}} = 2\left( {\frac{{\mathrm{D}}}{{\pi l^2}}} \right)^{1/2}$$

$${\mathrm{D}} = \frac{{s^2\pi l^2}}{4}$$

For absorption Mt = W_t_ – W_i_ and M_∞_ = W_s_ – W_i_

For desorption Mt = W_s_ – W_t_ and M_∞_ = W_s_ – W_d_

Where weight of hydrogels W_t_ = at time during swelling, W_i_ = initial weight, W_s_ = weight at equilibrium, W_d_ = dry weight after dehydration.

#### Tensile test

All hydrogel films were cut into dumbbell shapes and swollen in distilled water to equilibrium state before testing. The tensile tests were carried out at rate of 5 mm/min using a 50 KN load cell (Universal testing machine Instron 5569A).

#### Trouser tear test

This test method determines the force necessary to propagate a tear in the hydrogel films. Fracture energies were obtained using the trouser tear test on rectangular specimens with dimensions (50 × 17 mm) with a 33 mm long initial notch. The fracture energies of the hydrogels were calculated as

$${\mathrm{G}} = \frac{{2{\mathrm{Fave}}}}{{\mathrm{W}}}$$

Where F_ave_ is the average tearing force and W is the thickness of the samples.

#### Cytotoxicity evaluation of hydrogels

All test hydrogels were sterilised by gamma irradiation. Human osteoblasts (HOB) were expanded and used at passage 25. Cells were seeded at 1 × 10^5^cells/ml for indirect (elution) studies.

Cytotoxicity was determined using an elution study (MTT assay) at 24 and 48 h. Hydrogels were placed in DMEM cell culture media and placed on a roller for 24 and 48 h. Cells were cultured in the elution media at 37 °C in a CO_2_ incubator. Surviving HOB cells were quantified using MTT assay, the positive control group 10% alcohol in media and negative control cells in media were adopted.

Relative growth rate of the HOB cells was calculated from the average optical density (OD) values.

$$relative\,growth\,rate\,(RCR) = \left[ {\frac{{average\,of\,tested\,group\,OD \, - \, average\,of\,blank\,control\,OD}}{{\left( {{\mathrm{average}}\,{\mathrm{of}}\,{\mathrm{negative}}\,{\mathrm{control}}\,{\mathrm{OD} \, - \, \mathrm{average}}\,{\mathrm{of}}\,{\mathrm{blank}}\,{\mathrm{control}}\,{\mathrm{OD}}} \right)}}} \right] \times 100\%$$

Where: Non-cytotoxic >90% cell viability; Slightly cytotoxic = 60–90% cell viability; Moderately cytotoxic = 30–59% cell viability; Severely cytotoxic ≤30% cell viability.

#### Cell adhesion studies

Cells were cultured in the presence of hydrogels for 28 days, in cell culture medium before viability was analysed using a Live/dead viability/cytotoxicity kit (L-3224) from Invitrogen. For direct studies rolling method was used to seed hydrogels with 3.29 × 10^5^ cells for each sample, and 2 × 10^4^ cells/well for the controls. At each time point cells were incubated with 1 µM of Calcein AM and 2 µM of ethidium homodimer in PBS and placed in CO_2_ incubator for 20 min. Calcein stains the live cells green due to intracellular esterase activity, and ethidium stains the cells red as it enters cells with damaged membranes and becomes fluorescent upon binding to nucleic acids in the dead cell. The cells were imaged with a fluorescence microscope (Olympus IX51).

#### Scanning electron microscopy

Scanning electron microscopy was carried out on selected samples placed on aluminium stubs using conductive blue then coated in a thin layer of gold before being placed in a vacuum container of quanta field emission scanning electron microscope (Quanta 200F microscope (FEI), using 10–20 kV and a Magnification of 10,000×.

#### In vitro drug release

Hydrogels were loaded with a total of 1% vancomycin hydrochloride (Sigma Aldrich) to fabricate the drug containing hydrogels, by mixing a solution of PVA with 1% vancomycin at 300 rpm until it was homogenously incorporated in the solution. The PVA solution was allowed to settle to allow the release of all air bubbles before casting into moulds and subjecting to cycles of freeze thawing as previously described. For formation of the dual network hydrogels (DN), 1% vancomycin was added to the sodium alginate solution to prevent diffusion of the drug from PVA to sodium alginate. Once fully swollen with alginate the hydrogels were chelated with CaCl_2_ before subjecting to one more cycle of freeze drying.

Drug release studies were conducted in phosphate buffered saline solution (PBS), pH 7 at 37 °C. Dimensions of the specimens and weights were recorded before immersing in 2 ml PBS. 200 µl solution of samples was withdrawn at recorded time points and diluted with 500 µl distilled before reading on a UV spectrophotometer (Cecil 9000 series) at wavelengths 281 nm, the extracted 200 µl was replenished with exactly 200 µl at each time point. Tests were carried out in triplicates.

#### Statistical analysis

Statistical analysis was carried out using One way ANOVA with level of significance set at *p* < 0.05 for all calculations.

## Results and discussion

The dual networks were synthesized using poly (vinyl alcohol) solutions that were subjected to freeze-thawing cycles to obtain the first network through physical crosslinking. These xerogels were then equilibrated in dilute sodium alginate solution (2% w/v) and the second network formed through gelation of the alginate by using divalent calcium ions to obtain the dual networks. The networks were further subjected to one cycle of freeze thawing with the formulations shown in Table 1. Cryogelation was selected to physically cross link PVA since it is an attractive method of production of micro or macro porous materials [12] without use of crosslinking agents, initiators, activators or solvents that in addition allows control on textural, structural and absorption characteristics. Furthermore, it enables to vary the extent of crosslinks by altering factors such as the concentration of the macromolecules in solution and number of freeze-thawing cycles, which also allows a handle on altering the physical properties as per requirements. The use of naturally occurring polysaccharides such as sodium alginate have several advantages due to their biocompatibility and non-toxic properties coupled with the facile chelating conditions to render hydrogels. However, the resultant alginate gels usually lack mechanical strength and toughness thereby easily rupture on application of low stresses. The second network was thus formed once the aqueous solution of sodium alginate intruded the PVA hydrogel network and ionotropic gelation was conducted using calcium chloride at room temperature, thereby avoiding any toxic conditions, especially an advantage for biomedical applications. The networks were finally subjected to one more cycle of FT to enable any intermolecular interactions between the two polymers.

**Table 1:** Percentage of sodium alginate absorbed by the PVA hydrogels formed by different numbers of freeze-thawing cycles (*n* = 3)

Concentration of PVA (%)	Cycles of freeze thawing of PVA	% Sodium alginate absorbed in PVA hydrogel at equilibrium	% of alginate in the hydrogel after one cycle freeze thawing
10	1	76.32 ± 1.48	39.64 ± 0.56
	2	75.44 ± 0.44	34.91 ± 0.21
	3	74.73 ± 1.61	33.87 ± 2.31
20	2	63.58 ± 1.13	23.29 ± 0.36

Mass of PVA xerogels was measured before and after swelling to equilibrium in sodium alginate as well as after freeze drying, values of which were used to calculate percentage of alginate in the networks

To evaluate the effect of concentration of PVA, 10 and 20% w/v solutions were used in the study, however for the PVA 20 dual networks only 2 FT cycles were used, based on the results of the PVA 10 gels that did not exhibit differences in sodium alginate uptake between 2 and 3 FT cycles. Since the freeze drying process imbibes porosity in the PVA first network, a substantial swelling of the second network component is observed, which is further improved due to the ionizable carboxylate groups present in sodium alginate. The mass of PVA xerogels was measured before and after swelling to equilibrium in sodium alginate as well as after freeze drying, values of which were used to calculate percentage of alginate in the networks.The average overall amount of alginate present was found to range between 36% and 23% respectively for PVA 10 and 20 dual networks (Table 1). This also demonstrates the difference between the DbN and DN hydrogels as with the DN hydrogels the second polymer SA is of a much lower concentration in the final hydrogel network than the first PVA network.

### ATR-FTIR spectra of the dual network hydrogels

**Fig. 1:**
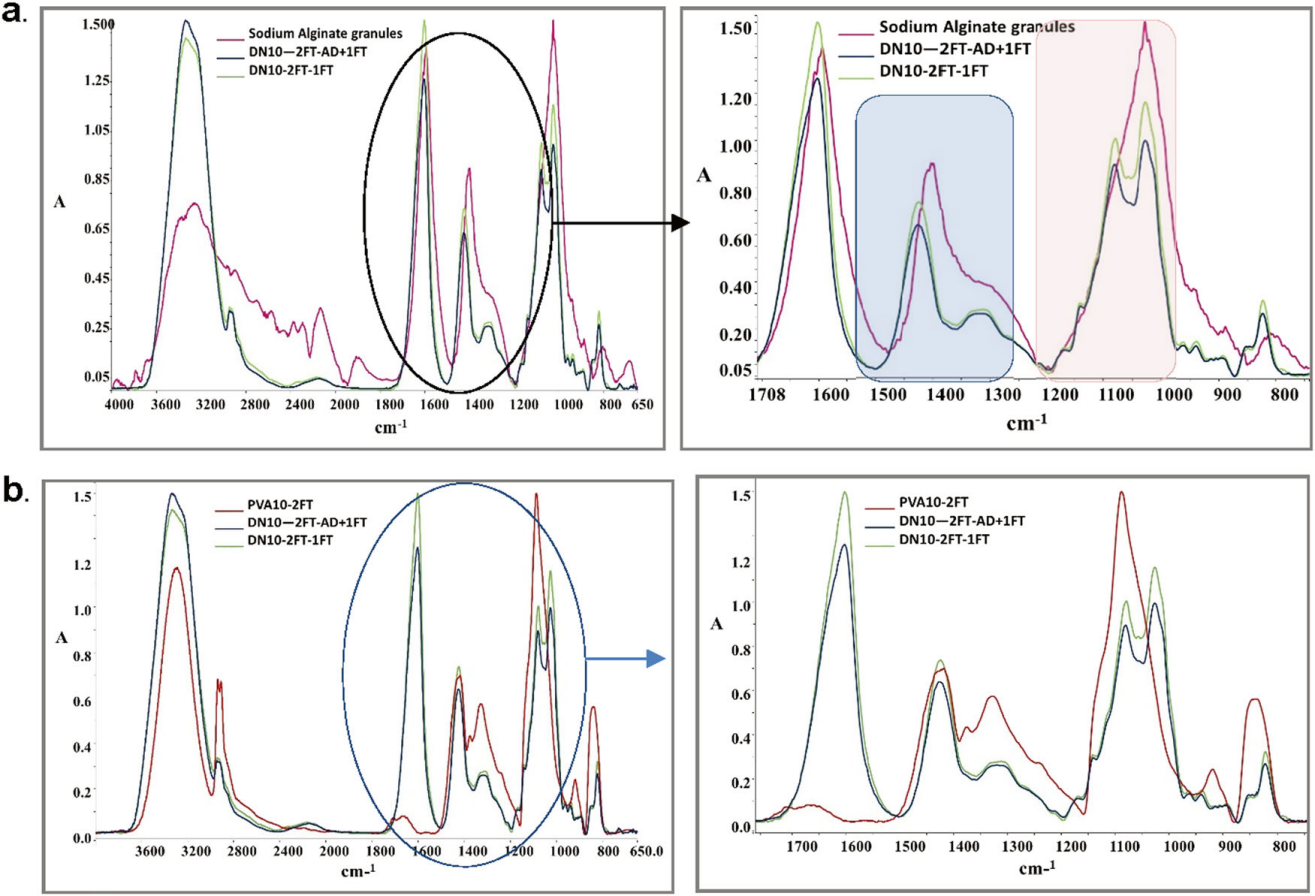
**a** An overlay of the FTIR spectra of sodium alginate granules, and dual network hydrogels chelated with calcium chloride and subjected to further crosslinking. **b** A comparison of infrared spectra (overlay) of PVA10-2FT, PVA10-2FT dual network hydrogels subjected to a further crosslinking of one freeze thaw cycle and air-drying prior to freeze thawing

The FTIR spectra (Fig. 1a) shows the ionic binding as calcium ions replaces the sodium ions in the alginate blocks, indicating calcium ions were able to penetrate and chelate the alginate within the PVA network. A comparison of the FTIR spectra of the dual networks with sodium alginate showed that a sharp -OH stretching peak (~3290 cm^−1^) increased in intensity as compared to the low broad peak of the SA granules due to the intramolecular H bonding in the dual network hydrogel. The -COO symmetrical peak (~1400 cm^−1^ highlighted in blue) in SA granules shifts to higher wavenumbers (~1420 cm^−1^) in the DN with a decrease in intensity due to the ionic binding, as the calcium ions replace sodium ions in the alginate blocks, therefore the charge density, radius and atomic weight of the cation are changed, creating a new environment around the carbonyl group. The highlighted region in blue shows the change in the peak arising at ~1300 cm^−1^, which becomes more pronounced in the DN as compared to sodium alginate, suggesting a strong binding of the calcium to the G blocks. The new peak at around 1150 cm^−1^ corresponds to a partial covalent bonding between calcium and oxygen atoms and the peaks at ~1070 and ~1026 cm^−1^ are associated with the guluronic blocks and indicate a change in the binding within these blocks as calcium is introduced [13].

The FTIR spectra of the dual networks also confirmed the presence of the alginate within the network due to the appearance of the ~1600 cm^−1^ carboxylate peaks of SA units. A slight shift in the carboxylate peak (Fig. 1b) highlighted in the blue region indicates an interaction between PVA and sodium alginate (the shift in the -COO^-^ peaks from 1418 to 1426 cm^−1^). Crosslinking between PVA and sodium alginate was indicated by the anti-symmetric stretch of the -C-OH in PVA forming a doublet peak in the dual network observed at 1080 and 1026 cm^−1^ revealing the presence of alginate within the structure as well as crosslinking of the PVA, indicating that there may be some interaction between the two polymers [14, 15].

### DSC-thermal analysis of the dual network hydrogels

The polymer-polymer interaction was evaluated by determining the glass transition temperature (Tg) of the networks. The Tg values of the dual networks ranged from 139–177 °C and showed a correlation to the number of FT cycles as expected. The single network of PVA exhibited a Tg at ~75 °C (Table 2) and this large difference from the dual networks also confirmed the formation of a new network that reinforces the matrix. The Tg values of the dual networks also showed a relationship with the concentration of PVA, with higher concentrations (20% PVA) yielding a lower Tg, which is attributed to the reduced uptake of alginate in the first network due to the higher crosslinking density of the PVA 20% network. The general increase in Tg of the dual networks due to the formation of tighter networks are in agreement with data reported on hydrogel membranes of SA and PVA by solvent casting, where a shift towards a higher temperature in the endothermic peaks of the hydrogels were reported [16]. Similarly the thermal stability of PVA-SA blends were reported to improve with inclusion of SA due to the hydrogen bonds between the carboxyl and hydroxyl groups of SA with hydroxyl groups of PVA [17] thus limiting chain movement during thermal treatment. The dual networks in this study were obtained either solely by a 2nd crosslinking of one cycle of freeze-thawing or subjected to air drying followed by freeze-thawing. The Tg values showed higher values for the latter, which can be attributed to the hydrogels incurring a combination of physical chain entanglements by air-drying in addition to hydrogen bonding that occurs on freeze drying of the network structures. The dual networks synthesized (DN20-2FT-1FT) using higher concentrations of PVA yielded lower Tg’s due to limited penetration of SA into the tight and dense network structure of PVA 20 single network hydrogel in comparison to the PVA10 networks.

**Table 2:** Glass transition, equilibrium water content, swelling ratio and gel fraction of PVA10 and PVA20 dual network hydrogels prepared by different cycles of freeze thawing (*n* = 3). (*x* = 1, 2 or 3 FT as shown in column 2)

Hydrogel network	Cycles of freeze thawing PVA	Glass transition, Tg (°C)	EWC (%)	Swelling ratio (SR)	Gel fraction, GF (%)	Diffusion coefficient, D, (10^−6^ cm^2^ s^−1^)
PVA10-(x)-1FT	2	73.4	81.3 ± 0.38	6.0 ± 0.13	89.0 ± 3.4	1.64
DN10-(x)-1FT	1	155.2 ± 3.0	69.1 ± 0.9	4.6 ± 0.1	70.6 ± 2.2	0.3
	2	163.6 ± 2.8	69.8 ± 0.5	4.7 ± 0.1	71.0 ± 1.3	0.1
	3	169.1 ± 3.2	71.8 ± 0.8	4.5 ± 0.1	78.4 ± 3.2	0.1
DN10-(x)-AD+1FT	1	169.3 ± 2.2	70.7 ± 1.4	4.7 ± 0.1	70.2 ± 1.2	0.4
	2	176.6 ± 2.6	72.6 ± 0.7	5.1 ± 0.1	72.1 ± 0.7	0.9
	3	177.4 ± 2.3	72.4 ± 0.7	4.7 ± 0.1	76.3 ± 1.0	0.8
PVA20-(x)-1FT	2	75.9	66.3 ± 0.7	3.0 ± 0.1	99.0 ± 1.7	1.0
DN20-(x)-1FT	2	139.0	55.0 ± 0.5	2.7 ± 0.01	83.4 ± 1.0	1.3

### Water uptake of the dual network hydrogels

Hydrogels placed in an aqueous environment allow the diffusion of water molecules that result in a swollen gel. The diffusion involves migration of water molecules into the network and affinity to water varies with composition and polymer architecture. The presence of the second network in the DN gels reduced the equilibrium water uptake (EWC) compared to the single network PVA hydrogels and this trend was reflected by a decrease from ~81.3% for the 10% PVA network to an average of ~70.2 and 71.9% for the dual network hydrogels with a second crosslinking of 1FT and AD+1FT respectively (Table 2). This decrease in EWC was found to be statistically significant (*P* < 0.001) indicating that addition of SA to the network structures followed by the different forms of second crosslinking resulted in hydrogels with a tighter network structure than the base single network PVA hydrogels, which would in turn lead to less swelling of the hydrogels in fluids due to the hindered mobility of polymer chains as compared to hydrogels with a loosely cross-linked network structure. In addition the higher concentration of PVA (PVA 20) used to create the single and double network exhibited lower EWC values than the 10% PVA networks due to the higher concentration of the hydroxyl groups, which is also similar to the trend reported for PVA-alginate blends [18]. The swelling ratio of the dual networks also decreased with addition of SA into the network structure, which can be attributed to the enhanced interaction between the SA and PVA [19]. Since SA is first ionically crosslinked with Ca^2+^ ions followed by further crosslinking by either 1FT or AD+1FT, it leads to an increase in the crosslink junctions resulting in lower EWC and SR. This trend was also reported by Hua et al. 2010 [14] for blends of PVA/SA beads that were crosslinked twice. Diffusion coefficient of the hydrogels indicated that there was limited migration of water molecules into the pre-existing spaces of the dual network hydrogels as compared to the PVA base hydrogel. These values (Table 2) decreased from an average of ~1.8 (10^−6^ cm^2^ s^−1^) in PVA to ~0.5 and 0.7(10^−6^ cm^2^ s^−1^) for the DN networks with 1FT and AD+1FT second crosslinking. The higher diffusion coefficient of AD-FT observed can be attributed to the fact that air drying prior to freeze thawing eliminates a large amount of water from the network hence these on FT do not form as many cross links as it would form between the two polymers if subjected to FT from the moist gel. Lower gel fractions were observed in PVA10 compared to PVA 20 as the higher concentration of hydroxyl groups lead to more effective gelling. The corresponding dual networks for PVA 10 and PVA 20 also contain the alginate network hence the reduction of gel fraction is attributed to the loss of unchelated and unreacted alginate within the network structure, this trend in results was found to be similar with results reported in a study by Kim et al. 2008 [17] on PVA alginate blends.

### Tensile strength of the dual network hydrogels

**Fig. 2:**
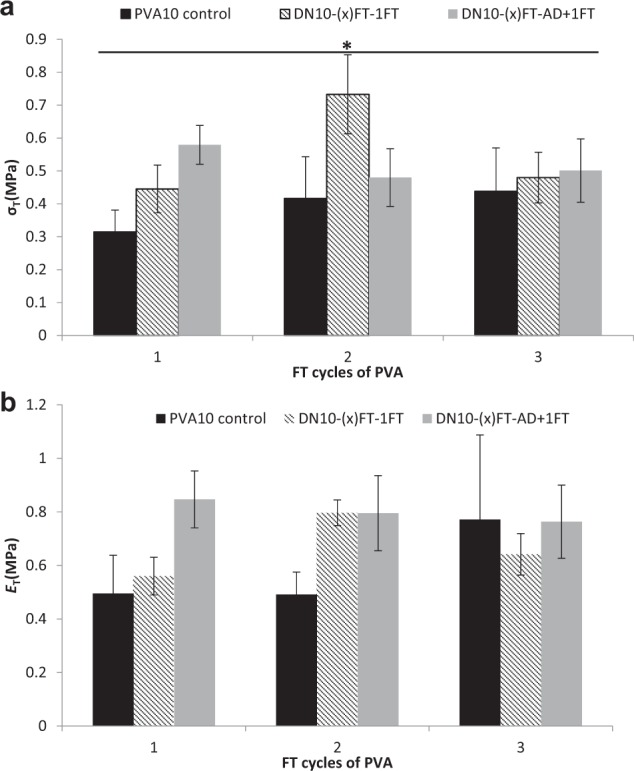
A comparison of **a** tensile strength and **b** Young’s modulus of the freeze-thawed PVA hydrogels with PVA/alginate dual network hydrogels prepared via various methods. (*n* = 6) (*P < 0.001); asterisk linked with lines indicate significant difference in values between the groups

Results (Fig. 2) show that crosslinking via AD+1FT did not significantly enhance the tensile strength as compared to only 1FT crosslinking. Analysis on the effect of number of FT cycles within each group of the individual groups (DN-1FT, DN-AD+1FT), showed that PVA and DN-AD+1FT hydrogels were found to have tensile strength values with no significant difference between freeze thaw cycles within each group, whereas two FT cycles of DN-1FT hydrogels yielded significantly higher (*P* < 0.001) tensile strength (0.7 MPa) than all the hydrogels. It would be expected that increasing the number of freeze thaw cycles (degree of crosslinking in the networks) would result in higher ultimate tensile strength, however it was evident from the results that a higher degree of crosslinking i.e. cycles of FT greater than 2FT yielded a more brittle network structure, hence lowered UTS. PVA-alginate blends for wound dressings have been reported by Kim et al. 2008 [17] where they found the addition of SA into blends resulted in decrease in tensile strength of their hydrogels with UTS values of 0.07 MPa with *E* of ~0.009 MPa for 5%(w/w%) and 3%(w/v) of SA, that are a magnitude of order lower than DN networks but exhibiting a similar trend. Xie et al. 2012 [19] reported a similar observation with a drop in tensile strength of their PVA-SA blends with addition of SA to PVA. Their highest recorded tensile strength with blends containing 25% SA with 4FT cycles was 0.2 MPa. A comparison of the blends with the dual networks also clearly indicate that the formation of a dual network has a significant effect on the tensile properties and it is the first brittle network that has a bearing on the tensile properties as is evident with the higher concentration of PVA. Kulkarni et al. 2010 [16] formed IPN’s of PVA and SA for drug release and found that tensile strength improved with the formation of IPN networks with maximum UTS values of ~0.4 MPa, whilst Gnanaprakasam et al. 2013 whose study on growth and survival of cells on biosynthetic PVA and SA reported a similar trend with tensile values of semi IPN and IPN hydrogel network of 0.8 MPa (disodium hydrogen phosphate and Ca^2+^ crosslinks) and 1.2 MPa (3% glutaradehyde crosslink) respectively [15].

From the current study it was apparent that PVA10-2FT hydrogels yielded dual network hydrogels with superior mechanical properties only with a second crosslinking of 1FT. To obtain dual network hydrogels with tensile strength greater than 1 MPa, PVA of concentration 20%(w/v) was used for 2FT cycles of freeze-thawing to form DN20-2FT-1FT.

**Fig. 3:**
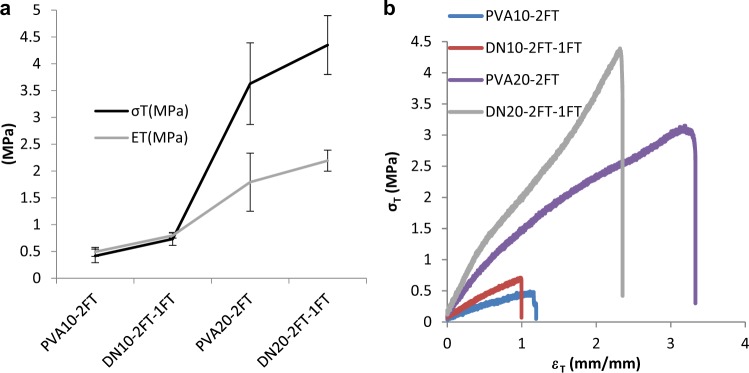
A comparison of **a** tensile strength and Young’s modulus, as well as (**b**) stress strain curves of the PVA (10, 20)-2FT and DN (10, 20)-2FT-1FT hydrogels. (*n* = 6)

The results (Fig. 3a) showed that tensile strengths of the dual network hydrogels could be increased by increasing the concentration of PVA to 20%, which were three orders of magnitude higher than most hydrogels. The modulus values of the dual networks indicated that they were dependent on the initial concentration of the first network, namely PVA. It can also be extrapolated that the stiffness of the single network hydrogels increases when SA is included in the network to form an IPN dual network. Tensile strength values obtained for DN20-2FT hydrogels were significantly higher than those reported in literature [15, 16]. We demonstrate from the stress-strain curves in tension mode (Fig. 3b) the ductile nature of the hydrogels illustrating the ability to undergo strains of ~≥1 mm/mm. The stress strain curves (Fig. 3b) indicate the ductility of the hydrogels proving to have strains >2 mm/mm.

### Fracture toughness of the dual network hydrogels

Tear tests are used as a measurement of fracture energy to qualify the mechanical strength of hydrogels. Fracture properties are related to specific material parameters such as critical fracture toughness, energy release rate, fracture energy and crack propagation resistance, which can be determined using a fracture mechanical test method such as a trouser tear test. Tearing energy includes surface energy, energy dissipated in plastic flow processes, and energy dissipated irreversibly in viscoelastic processes.

**Fig. 4:**
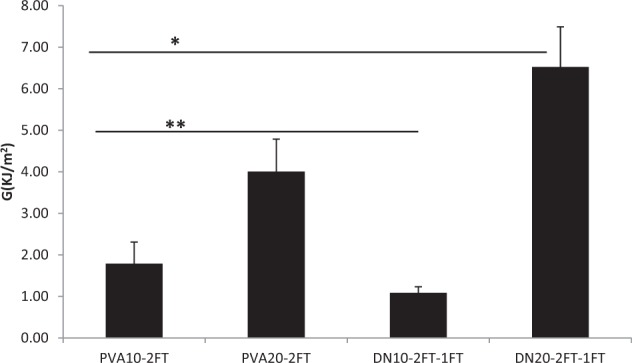
Fracture energy values of PVA10, 20-2FT and dual network hydrogels obtained from using a trouser tear test. (*n* = 6); asterisk linked with lines indicate significant difference in values between the groups

Fracture energies of the hydrogels (Fig. 4) showed that PVA20 dual network hydrogel had a significantly higher (*P* < 0.001) fracture energy of 6.5 KJ/m^2^ in comparison with all the other hydrogels. The increase in concentration of PVA lead to significantly higher strengths as well as incorporation of SA into the PVA base network structure. The opposite result however was observed with the PVA10 dual network where a lower fracture energy was obtained than its parent base PVA10 hydrogel. This could be attributed to the fact that more alginate is absorbed in the PVA10 network structure as compared to the PVA20, and with alginate being weaker than PVA in turn resulted in a decreased fracture toughness. Fracture toughness energies obtained in this study were compared with those obtained from double network hydrogels and the toughness values of the dual networks were ~≥2 fold than those reported in literature [20, 21].

### SEM analysis of the dual network hydrogels

**Fig. 5:**
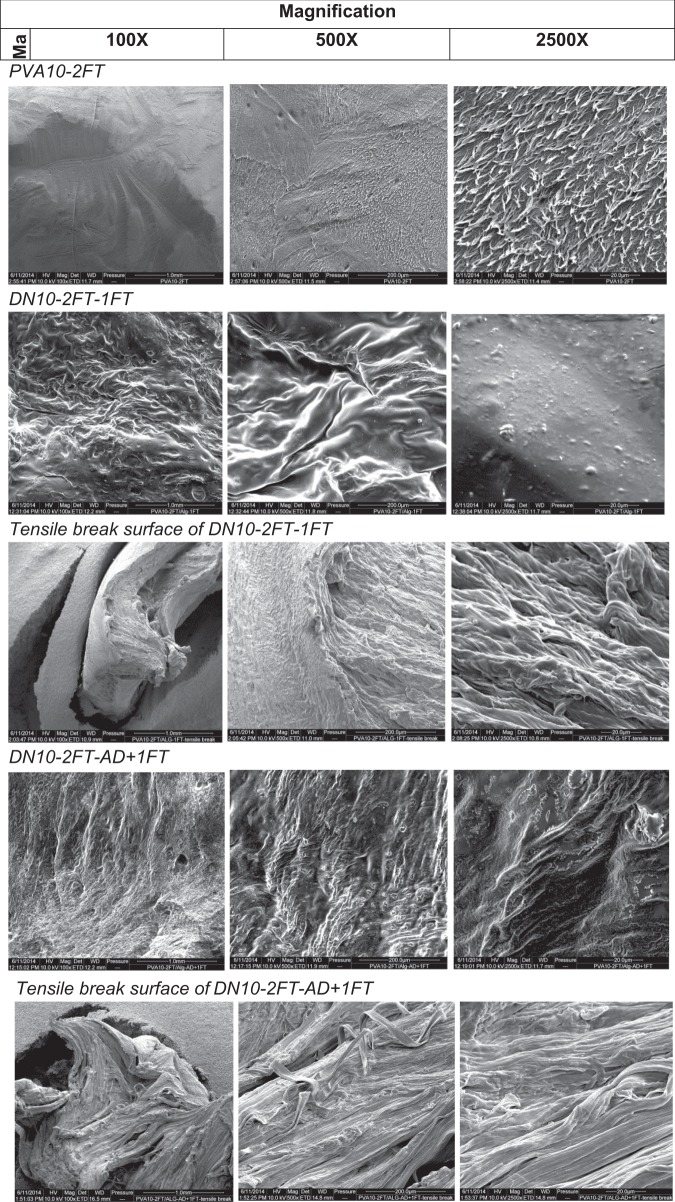

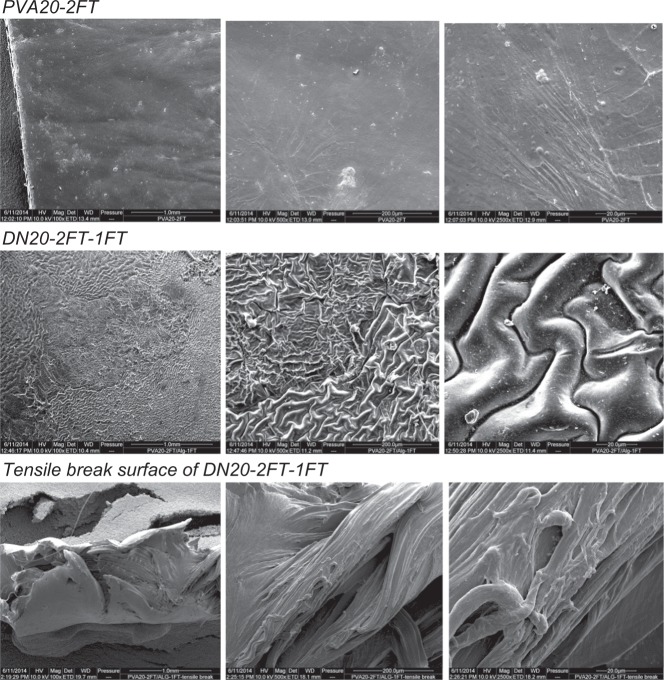
SEM micrographs of PVA10-(1,2,3)FT, DN(10,20)-2FT-1FT, DN-2FT-AD+FT, at different magnifications 100×, 500× and 2500×, as well as tensile break surfaces of the dual network hydrogels after tensile testing, indicating the different morphologies

Figure 5 shows the micrographs of PVA10-2FT xerogels with a microporous structure on the surface that appears to be crystallite domains, whereas the PVA20-2FT xerogel exhibit a dense smooth surface with little to no visible porosity, which is in agreement with the low EWC and SR results. The dual network hydrogels had a rough non-ordered surface area, which was attributed to the presence of alginate domains within the PVA network structure, also confirmed from the FTIR spectral data. The micrographs of the fractured surfaces of the tensile specimens show stretched elongated fibres of the hydrogels indicating that the hydrogels are ductile, also confirmed by tensile test results shown in Fig. 3b.

The dual networks in contrast show a fibrillar morphology, with entwined fibrilS and a difference in the morphology is observed within the network. The fracture tensile surfaces of the dual networks indicate a ductile fracture compared to PVA networks that show a brittle fracture. This observation is in agreement with results obtained from tensile test where strains of the hydrogels were >1 mm/mm.

**Fig. 6:**
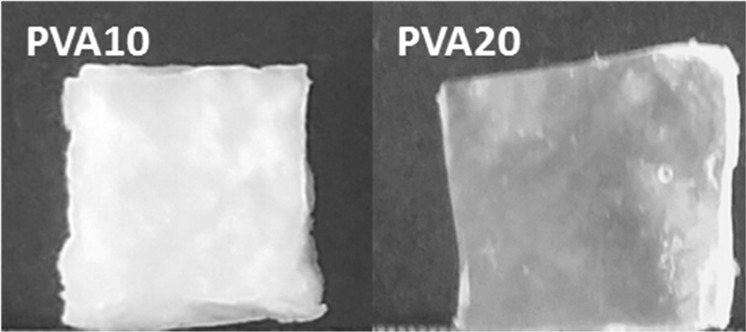
A photographic image of freeze thawed PVA hydrogels with 10 and 20% w/v concentration, demonstrating the difference in final appearance of the hydrogels

There were also distinct differences in the appearance of the PVA10 and 20 hydrogels, which may not be clearly visible in the SEM images but is demonstrated in photographic images shown in Fig. 6. The lower concentrations of PVA yielded more opaque hydrogels whilst PVA20 hydrogels were translucent, such an observation has also been reported in a previous study [22]. This variation in opacity arises due to the difference in solution viscosity, since lower concentrations of the polymer solution imposes less restriction on the movement of the PVA chains thereby making it easier for them to crystallise more effectively. This results in a hydrogel with large crystallites that scatter more visible light, which makes the hydrogels appear opaque. An increase in PVA concentration results in an aqueous solution with high viscosity and more PVA chains to form crystallites, however the crystallite size decreases as there is restricted growth and therefore scatters less visible light, which results in translucent hydrogels [22].

### Cytocompatibility

**Fig. 7:**
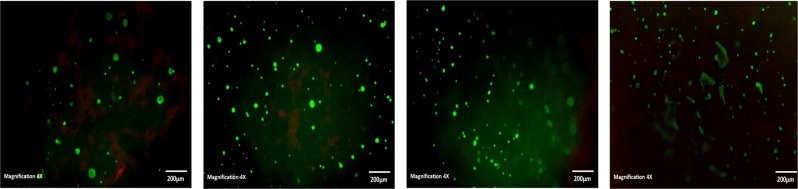
Live/dead staining of the HOB cells cultured on the PVA10-2FT hydrogel at day 3, 7, and 28 days (×4 magnification) showed cell attachment and proliferation on all the scaffolds. (Green: live cells and Red: dead cells)

**Fig. 8:**
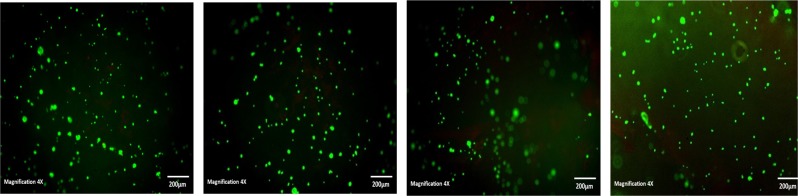
Live/dead staining of the HOB cells cultured on the DN10-2FT-1FT hydrogel hydrogel at day 3, 7, 14 and 28 days (×4 magnification) showed cell attachment and proliferation on all the scaffolds. (Green: live cells and Red: dead cells)

The degradation products of the PVA hydrogels were found to be slightly toxic increasing to moderately toxic at 48 h exposure (Table 3). These results are contradictory to those obtained in a study by Gnanaprakasam et al. 2013 [15], where they reported a higher viability of fibroblast cells on their hydrogel extracts indicating that the degradation products for their PVA-alginate blends were not toxic. However, the DN network eluants both at 24 and 48 h when exposed to HOB cells for 24 h exhibited a higher relative growth rate (Table 3) but prolonged exposure for 48 h yielded a slight lowering in percentage RGR. Nevertheless, the live/dead staining over a period of 28 days (Figs. 7 and 8) showed that HOB cells were able to migrate from the surface into the internal structure of the hydrogels. Cell were observed at different depths within the hydrogels illustrating and confirming the ability of the hydrogels to not only present as biocompatible, with good cell adhesion and attachment, but also indicating the ability of the cells to migrate and proliferate within the hydrogel networks. Earlier time point observation (day 7) of cell morphology showed cells aggregating to form bollus shape cells that later spread out along the surface of the hydrogel, beginning to form a sheet like structure over the surface. Literature reports that surface aspects of a biomaterial such as, topography, chemistry and surface energy determine cell behaviour upon contact. Cells in contact with a surface will first attach, adhere and spread, the quality of adhesion will influence their morphology and their capacity to proliferate and differentiate [23]. At 28 days in culture the live/dead images of the dual networks present the formation of a sheet of cells covering the surface of the hydrogel network.

**Table 3:** Relative growth rate percentage of HOB cells on PVA10-2FT and DN10-2FT-1FT demonstrating viability of HOB cells within the hydrogels

Hydrogel system	Eluents collected time (h)	Relative growth rate (RGR)(%)	
		24 h exposure	48 h exposure
PVA 10-2FT	24	65.6	45.4
	48	63.5	45.2
DN10-2FT-1FT	24	98.2	58.9
	48	75.9	50.7

### In vitro drug release of the dual network hydrogels

The dual networks developed exhibit properties that are potentially suited as scaffolds for soft and hard tissue engineering, wound healing and dressing applications, hence a preliminary study of these dual networks as potential drug carriers was evaluated using vancomycin, a widely used antibiotic. Vancomycin is a commonly effective antibiotic, recommended for chronic wounds, including those with moderate to severe osteomyelitis. It is highly effective against gram positive bacteria and used in hospital epidemics as a first line of defence against deadly resistant streptococcal and staphylococcal strains such as *Staphylococcus aureus*, which are becoming resistant to penicillin, methicillin and other β−lactam antibiotics [24–26].

### Drug and polymer Interaction

**Fig. 9:**
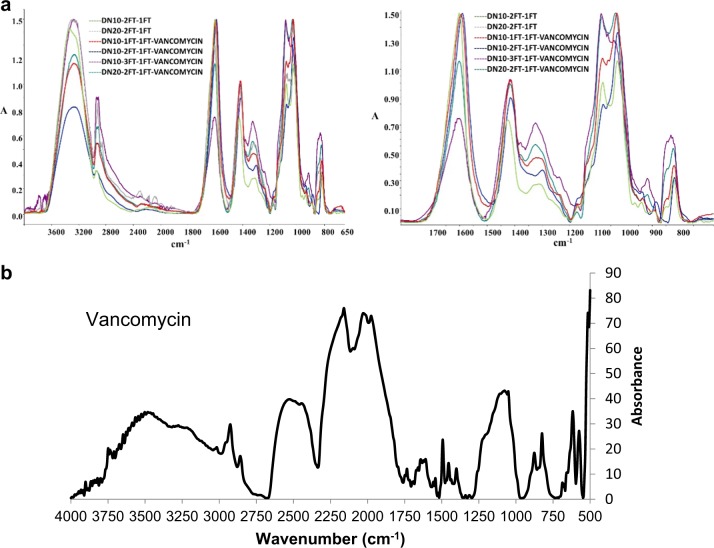
**a** An overlay comparison of infrared absorbance peaks of dual network hydrogels incorporated with and without vancomycin, subjected to different cycles of freeze thawing. **b** *An FTIR spectrum of vancomycin*

Vancomycin hydrochloride hydrate (Mw 1485.71 anhydrous basis) has a complex structure consisting of a seven membered peptide chain linked to two unique sugar moieties. The drug is known to bind to peptides and polymer surfaces, as well as act at a chelator. Vancomycin has also been reported to dimerize with itself specifically due to amide-amide hydrogen bonding, hydrophobic and ionic interactions, therefore it is expected to form hydrogen bonds as well as elicit hydrophobic interactions with alginate units. The FTIR spectra of the drug and polymer scaffolds shown in Fig. 9, showed an increase in intensity of the peaks between 1700–1500 cm^−1^ due to amide groups of the vancomycin as well as 1500–1200 cm^−1^ was observed indicating the presence and incorporation of vancomycin in the base PVA network structure.

### In vitro drug release profile

**Fig. 10:**
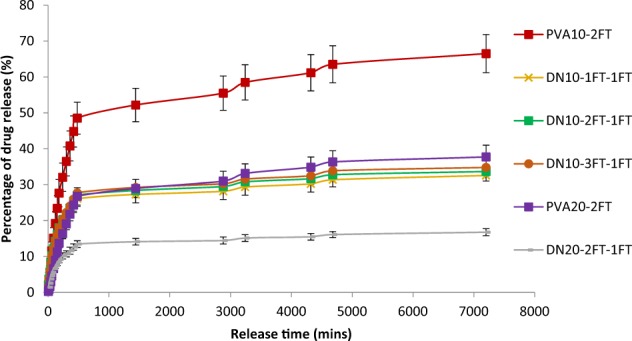
Percentage drug release as a function of time from the PVA-2FT only hydrogel and the dual network hydrogels. (*n* = 3)

**Fig. 11:**
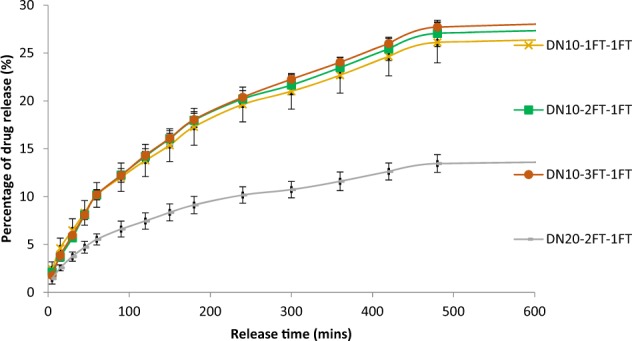
The vancomycin percentage release profile in the first 600 min as a function of time from the dual network hydrogels. (*n* = 3)

Figures 10 and 11 illustrate the release profile of entrapped vancomycin within the hydrogels. The dual networks exhibited a slower release in comparison to the single network with ~17–25% of the drug being released within the first 500 min and less than 40% of the drug being released after 7000 min.

A linear (high) release rate is observed with the PVA10 single network hydrogels with up to 59% vancomycin being released from the hydrogels within the first 8 h. This linear release is attributed to the porous structure of the PVA10 hydrogels attained during the sublimation process of water ice crystals within the network. This then in turn led to a large surface area of the hydrogel exposed to dissolution of the vancomycin drug into the PBS solution. Increase in concentration of PVA to 20% led to a decrease in total drug released (27%) after 500 min, due to the dense network structure of PVA formed with higher concentrations. However, the DNs exhibited a lower drug release rate with a maximum of ~27% after 500 min, which is attributed to the tight interpenetrating network structure attained in formation of the dual networks as well as reduced porosity within these networks, thereby hindering the transport of drug molecules through the membrane. It was observed that drug release rate was closely related to EWC and SR of the hydrogels, therefore the higher the swelling ratio of the hydrogel the higher the % drug release and vice versa. Similar observations have been reported in other studies, where Kulkarni et al [16] reported that their PVA hydrogels loaded with prazosin hydrochloride showed a maximum release of ~99.3% after 12 h, with a high release rate observed in the initial hours, where as their PVA/SA IPN membranes had an extended drug release for up to 24 h. They also reported that the drug release in their hydrogels decreased with increase in PVA concentration [16]. Hau et al reported that their dual crosslinked beads (chelated blend of PVA/SA subjected to 2 cycles of freeze thawing) showed a lower initial drug release profile of diclofenac sodium as compared to the PVA/SA CaCl_2_ crosslinked beads [14]. Variation of freeze thaw cycles in this study did not show a significant difference in drug release rates within the individual groups.

It has been reported that high initial linear drug release profiles are useful for immediate eradication of bacteria while slower release profiles are appropriate for preventing infection or recolonisation of the wound by bacteria [24]. Therefore the initial linear increase followed by slower drug release profile of the DN hydrogels would prove suitable for simultaneously eradicating an existing infection while preventing recolonisation of the wound by bacteria.

### Potential applications of the formed hydrogels in regenerative medicine

It is now common practise for surgoens to collect and spin the patients waste blood while in the operating theater, so as to collect PRP rich in growth factors and apply it at the wound site with the aim of enhancing healing and recovery time of the wound. Given the properties of the fabricated dual network hydrogels, various applications are proposed where the hydrogels can integrate with surrounding tissues and induce new tissue formation.

**Fig. 12:**
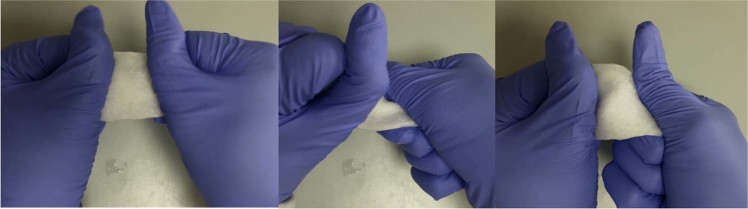
Picture images illustrating the nature and flexibility of the dual network hydrogels

Dual network hydrogels are porous tough flexible hydrogels (demonstrated in Fig. 12) that can be loaded with soluble signalling molecules such as bone morphogenetic proteins or platelet rich plasma as well as antibiotics, and be used in areas such as wound healing. Autologous PRP could be imbibed within the hydrogel before placing on the wound site so as to allow for localised delivery of growth factors and hence enhanced regeneration and recovery time of the wound. DN hydrogels have the versatility to be fabricated in different sizes shapes and thickness, therefore suited for skin substitution applications, the networks can be fabricated as thin films of which skin cells (keratinocytes, melanocytes and Langerhans cells) can be cultured onto before the skin substitution surgery. It can also be noted that to allow for enhanced cell attachment and proliferation, peptide-coupled alginates can be used for the formation of dual network hydrogels [27].

## Conclusion

In conclusion, we report the successful synthesis of tough interpenetrating dual network hydrogels of PVA and alginate using conditions that involve non toxic solvents or crosslinking agents suited for biomedical applications. These hydrogels are flexible, ductile and porous with the ability to absorb and retain fluids as well as can be easily used to incorporate drugs/antibiotics for the matrices to be applied as drug delivery systems. The dual network hydrogels can be tailored to have varying mechanical properties, shapes, size and thickness to suit the intended clinical application. These hydrogels hold great promise in biomedical applications such as wound healing and tissue regeneration, with an added advantage for further development in bone composite formulations, by incorporation with fillers.

## References

[1] Annabi N, Tamayol A, Ali J, Uquillas JA, Akbari M, Bertassoni LE, Cha C (2014). 25th anniversary article: rational design and applications of hydrogels in regenerative medicine. Adv Mater.

[2] Costa AMS, Mano JF (2015). Extremely strong and tough hydrogels as prospective candidates for tissue repair–a review. Eur Polym J.

[3] Gyles DA, Castro LD, Silva Jr OC, Ribeiro-Costa RM (2017). A review of the designs and prominent biomedical advances of natural and synthetic hydrogel formulations. Eur Polym J.

[4] Boateng JS, Matthews KH, Stevens HNE, Eccleston GM (2008). Wound healing dressings and drug delivery systems: a review. J Pharm Sci.

[5] Lay-Flurrie K (2004). The properties of hydrogel dressings and their impact on wound healing. Prof Nurse.

[6] Courtney T, Sacks MS, Stankus J, Guan J, Wagner WR (2006). Design and analysis of tissue engineering scaffolds that mimic soft tissue mechanical anisotropy. Biomaterials.

[7] Miserez A, Weaver JC, Chaudhuri O (2015). Biological materials and molecular biomimetics—filling up the empty soft materials space for tissue engineering applications. J Mater Chem B.

[8] Ahmed S, Nakajima T, Kurokawa T, Haque A, Gong JP (2014). Brittle–ductile transition of double network hydrogels: mechanical balance of two networks as the key factor. Polymer.

[9] Gong JP (2010). Why are double network hydrogels so tough?. Soft Matter.

[10] Nakajima T, Sato H, Zhao Y, Kawahara S, Kurokawa T, Sugahara K, Gong JP (2012). A universal molecular stent method to toughen any hydrogels based on double network concept. Adv Funct Mater.

[11] Liu XJ, Ren XY, Guan S, Li HQ, Song ZK, Gao GH (2015). Highly stretchable and tough double network hydrogels via molecular stent. Eur Polym J.

[12] Nkhwa S, Lauriaga KF, Kemal E, Deb S (2014). Poly(vinyl alcohol): physical approaches to designing biomaterials for biomedical applications. Conf Pap Sci.

[13] Sartori C, Finch DS, Ralph B, Gilding K (1997). Determination of the cation content of alginate thin films by FTIR spectroscopy. Polymer.

[14] Hua S, Ma H, Li X, Yang H, Wang A (2010). pH-sensitive sodium alginate/poly(vinyl alcohol) hydrogel beads prepared by combined Ca^2+^ crosslinking and freeze-thawing cycles for controlled release of diclofenac sodium. Int J Biol Macromol.

[15] Gnanaprakasam Thankam F, Muthu J, Sankar V, Gopal RK (2013). Growth and survival of cells in biosynthetic poly vinyl alcohol–alginate IPN hydrogels for cardiac applications. Colloids Surf B Biointerfaces.

[16] Kulkarni RV, Sreedhar V, Mutalik S, Setty CM, Sa B (2010). Interpenetrating network hydrogel membranes of sodium alginate and poly(vinyl alcohol) for controlled release of prazosin hydrochloride through skin. Int J Biol Macromol.

[17] Kim JO, Park JK, Kim JH, Jin SG, Yong CS, Li DX (2008). Development of polyvinyl alcohol-sodium alginate gel-matrix-based wound dressing system containing nitrofurazone. Int J Pharm.

[18] Chhatri A, Bajpai J, Bajpai AK, Sandhu SS, Jain N, Biswas J (2011). Cryogenic fabrication of savlon loaded macroporous blends of alginate and polyvinyl alcohol (PVA). Swelling, deswelling and antibacterial behaviors. Carbohydr Polym.

[19] Xie L, Jiang M, Dong X, Bai X, Tong J, Zhou J (2012). Controlled mechanical and swelling properties of poly(vinyl alcohol)/sodium alginate blend hydrogels prepared by freeze–thaw followed by Ca^2+^ crosslinking. J Appl Polym Sci.

[20] Nakajima T, Furukawa H, Tanaka Y, Kurokawa T, Osada Y, Gong JP (2009). True chemical structure of double network hydrogels. Macromolecules.

[21] Tanaka Y, Kuwabara R, Na YH, Kurokawa T, Gong JP, Osada Y (2005). Determination of fracture energy of high strength double network hydrogels. J Phys Chem B.

[22] Hong H, Liao H, Chen S, Zhang H (2014). Facile method to prepare self-healable PVA hydrogels with high water stability. Mater Lett.

[23] Anselme K (2000). Osteoblast adhesion on biomaterials. Biomaterials.

[24] Shukla A, Avadhany SN, Fang JC, Hammond PT (2010). Tunable vancomycin releasing surfaces for biomedical applications. Small.

[25] Hernandez R (2006). The use of systemic antibiotics in the treatment of chronic wounds. Dermatol Ther.

[26] Schäfer M, Schneider TR, Sheldrick GM (1996). Crystal structure of vancomycin. Structure.

[27] Andersen T, Strand BL, Formo K, Alsberg E, Christensen BE (2012). Alginates as biomaterials in tissue engineering. Carbohydr Chem.

